# Effect of a multicomponent behavioural PMTCT cluster randomised controlled trial on HIV stigma reduction among perinatal HIV positive women in Mpumalanga province, South Africa

**DOI:** 10.1080/17290376.2018.1510787

**Published:** 2018-08-22

**Authors:** Karl Peltzer, Suat Babayigit, Violeta J. Rodriguez, Jenny Jean, Sibusiso Sifunda, Deborah L. Jones

**Affiliations:** a HIV/AIDS/STIs and TB (HAST) Research Programme, Human Sciences Research Council, Pretoria, South Africa; b Department of Research & Innovation, University of Limpopo, Sovenga, South Africa; c Department of Psychiatry & Behavioral Sciences, University of Miami Miller School of Medicine, Miami, Florida, USA; d Department of Psychology, University of Georgia, Athens, Georgia, USA; e University of Rochester, Rochester, New York, USA; f Department of Psychiatry, University of Zambia School of Medicine, Lusaka, Zambia

**Keywords:** Randomized controlled trial, behavioural intervention, stigma, Prevention of Mother to Child Transmission of HIV (PMTCT), South Africa

## Abstract

***Background:*** We evaluate the impact a multicomponent, behavioural, prevention of mother to child transmission (PMTCT), cluster randomised controlled trial on HIV stigma reduction among perinatal HIV infected women in rural South Africa. ***Methods:*** In a cluster randomised controlled trial, twelve community health centres (CHCs) in Mpumalanga Province, South Africa, were randomised; pregnant women living with HIV enrolled received either: A Standard Care (SC) condition plus time-equivalent attention-control on disease prevention (SC; 6 CHCs; *n* =357), or an Enhanced Intervention (EI) condition of SC PMTCT plus the ‘Protect Your Family’ intervention (EI; 6 CHCs; *n* =342). HIV-infected pregnant women in the SC attended four antenatal and two postnatal video sessions; those in the EI, four antenatal and two postnatal group PMTCT sessions, including stigma reduction, led by trained lay health workers. Maternal PMTCT, HIV knowledge and HIV related stigma were assessed. The impact of the EI was ascertained on stigma reduction (baseline, 12 months postnatally). A series of logistic regression and latent growth curve models were developed to test the impact of the intervention. ***Results:*** In all, 699 women living with HIV were recruited during pregnancy (8–24 weeks), and assessments were completed prenatally at baseline and at 12 months (59.5%) postnatally. Baseline scores of overall HIV related stigma and the four scale factors (personalised stigma, disclosure concerns, negative self-image, and concern public attitudes) decreased at follow-up in the intervention group, while baseline scores of overall stigma and three scale factors (personalised stigma, negative self-image, and concern public attitudes) increased at follow-up in the control group. Using longitudinal analyses, Model 1, which included time-invariant predictors of stigma assessed over the two time periods of baseline and 12 months, increases in stigma from baseline to 12 months were associated with being unemployed, having been diagnosed with HIV before the current pregnancy, and alcohol use. In Model 2, which included time-varying predictors, lower stigma scores were associated with participation in the intervention, greater male partner involvement, and consistent condom use. ***Conclusion:*** The enhanced PMTCT intervention, including stigma reduction, administered by trained lay health workers had a significant effect on the reduction of HIV related stigma. ***Trial registration:*** clinicaltrials.gov: number NCT02085356

## Introduction

Stigma related to HIV infection remains a pervasive issue in South African communities, particularly among pregnant women. Antiretroviral therapy (ART) initiation, adherence, and retention in care among these women have all been found to be negatively impacted by HIV-related stigma (Hodgson et al., 2014). Many women living with HIV (WLHIV) fear the stigmatisation that may result from receiving treatment in HIV clinics. The long wait times at clinics may increase HIV-associated visibility for women and generate feelings of being judged (Awiti-Ujiji et al., 2011), and those obtaining ART may do so under the guise of pregnancy medications (Clouse et al., 2014). Fears of negative consequences related to stigma often lead WLHIV to avoid disclosing their HIV status (Crankshaw et al., 2014; Demmer, 2011; Hodgson et al., 2014), and women may wait until their child is born, tested for HIV, and found to be infected to disclose their own HIV status (Crankshaw et al., 2014). WLHIV face being blamed, divorced or abandoned by their partner, and the loss of vital familial income (Demmer, 2011; Hodgson et al., 2014). WLHIV may also fear doubly disappointing their families, once due to the pregnancy and again due to their HIV status (Crankshaw et al., 2014). Impacting the health of WLHIV, especially when pregnant, the reduction of stigma among WLHIV and the communities in which they exist is essential.

Stigma-related interventions have utilised different mechanisms and approaches, e.g. increased education on HIV, the effectiveness of treatment, and the harmful effects of stigma (UNAIDS Report on the Global AIDS Epidemic, 2010), provision of social support and increasing self-esteem (Kotze, Visser, Makin, Sikkema, & Forsyth, 2013), community level interventions (Brittain et al., 2017), integration of HIV services and counselling (Crankshaw et al., 2014), and integrating HIV and maternal/child health services (Hodgson et al., 2014).

Much of the systematic reviews analyzing the effect of interventions aimed at reducing public stigma attitudes towards people living with HIV (PLHIV) have demonstrated some level of reduction in stigma about PLHIV (Mak, Mo, Ma, & Lam, 2017; Sengupta, Banks, Jonas, Miles, & Smith, 2011). Few studies have evaluated HIV stigma reduction interventions with PLHIV. In a group-based pre–post design behavioural intervention, including stigma, for HIV positive youth in four developing countries, HIV-related stigma (personalised stigma, disclosure concerns, and negative self-image) reduced, but were largely not maintained at three months follow-up (Harper, Lemos, & Hosek, 2014). A stigma reduction intervention (video on women with HIV infection) trial among HIV-infected women living in Southern USA achieved a large intervention effect in reducing overall stigma in both intervention and control groups (Barroso et al., 2014). A study conducted by Uys et al. (2009), examining the impact of an interventional protocol addressing stigma among PLHIV and nurses demonstrated significant reduction in HIV-related stigma and increased self-esteem among PLHIV but not among the nurses. In a qualitative study in South Africa, a community HIV stigma-reduction intervention led stigma reduction in PLHIV (French, Greeff, Watson, & Doak, 2015).

Although interventions can reduce stigma among PLHIV, healthcare workers and families of PLHIV, the mode by which stigma is assessed and lack of internal validity of some measures adds to the challenge of determining their efficacy (Li, Liang, Lin, Wu, & Rotheram-Borus, 2010; Sengupta et al., 2011; Uys et al., 2009). Overall, interventions with higher numbers of sessions appear to result in greater reductions in stigma, post intervention (Mak et al., 2017). Studies addressing uptake and adherence to prevention-mother-to-child (PMTCT) protocols emphasise the role of male partners (Farquhar et al., 2004; Koo, Makin, & Forsyth, 2013), and the importance of reducing stigma to promote men’s engagement in the PMTCT process (Mahajan et al., 2008) and enhance women’s HIV disclosure (Mahajan et al., 2008; Makin et al., 2008). Stigma reduction may increase men’s willingness to engage in HIV testing and increase women’s comfort in inviting partners to attend clinic visits during pregnancy (Katz et al., 2009).

This study sought to longitudinally examine the impact of an intervention on HIV-related stigma among South African HIV-infected pregnant women, both newly and previously diagnosed. It was theorised that an intervention addressing both PMTCT and stigma reduction could decrease stigma during the perinatal period.

## Methods

### Study design

Study data was drawn from an ongoing longitudinal, clinic-randomised, PMTCT controlled trial, conducting a baseline assessment prenatally (8–24 weeks), and a long-term follow up assessment postnatally (12 months). The trial was aimed to increase PMTCT uptake in 12 rural community health centres (CHCs) in Gert Sibande and Nkangala districts in Mpumalanga province, South Africa (Jones et al., 2014). Clinics were randomised to a Standard Care (SC) condition plus time-equivalent attention-control on disease prevention (SC; 6 CHCs) or an Enhanced Intervention (EI) condition of SC PMTCT plus the ‘Protect Your Family’ intervention (EI; 6 CHCs).

Enhanced Intervention (EI) condition. The EI condition consisted of standard care PMTCT counselling by nursing staff during perinatal care plus the ‘Protect Your Family’ intervention, which was comprised of four antenatal and two postnatal group PMTCT sessions led by trained lay health workers. Sessions addressed HIV knowledge, prevention of vertical transmission, adherence to PMTCT and medication use, HIV testing, prevention of HIV transmission and stigma, HIV disclosure, communication with partners, intimate partner violence (IPV), infant feeding, safer conception, family planning and dual method sexual barrier use. The stigma component of the intervention was presented in sessions one and two, in the context of communication with partners, HIV status disclosure and medication adherence. During sessions, participants received cognitive behavioural skill training addressing the key components of each session, e.g. the impact of stigma-related thoughts on perceptions and behaviours. Group members were encouraged to problem solving, provision of supportive feedback and peer mentorship on session topics, role-playing communication strategies and negotiation. Homework addressed strategies practiced in the sessions (Jones et al., 2014).

Standard Condition (SC). The control condition consisted of standard care PMTCT counselling by nursing staff during perinatal care plus time-equivalent attention-control videos. Videos were presented to participants in a group-administered session and addressed childhood disease prevention, 1) diarrhea management, dehydration and breast feeding, 2) infant nutrition, 3) immunisation and sexual abuse, and were then followed by one individual or couples session on 4) fevers, and post-partum, two couples or individual video sessions on 5) burns, 6) alcohol use. Presentations were supervised by a lay health worker (Jones et al., 2014).

### Sample and procedure

Eligible women were HIV-seropositive pregnant women with partners, between 8 and 24 weeks pregnant, the typical time of entry into antenatal care, and aged 18 years or older. Following provision of written informed consent, all women completed study measures in their preferred language (English, isiZulu, or seSotho) using ACASI to enhance disclosure and reduce interviewer bias. To familiarise participants with the computer system, assessors completed the demographic component of the questionnaire together with participants, followed by participant-only completion of all other assessments. In addition, an on-site assessor was available to assist where necessary and answer any questions.

Ethical approval was granted by the Human Sciences Research Council (HSRC) Research Ethics Committee (REC), protocol approval number REC4/21/08/13. Study approval was also obtained from the Department of Health and Welfare, Mpumalanga Provincial Government, South Africa and the University of Miami Miller School of Medicine Institutional Review Board (IRB ID: 20130238), and the study was registered as a clinical trial on clinicaltrials.gov, number NCT02085356. All participants provided written informed consent.

## Measures

*The HIV Stigma Scale,* consisting of 40 items, was used as an overall scale, measuring four dimensions of stigma: (1) personalised stigma (18 items), (2) disclosure concerns (8 items), (3) negative self-image (8 items) and (4) concerns with public attitudes (8 items) (Response options ranged from 1 = completely disagree to 4 = completely agree) (Berger, Ferrans, & Lashley, 2001). Cronbach alpha for the overall scale was 0.96 at baseline (sub-scales: personalised stigma 0.94, disclosure concerns 0.79, negative self-image 0.81 and concern public attitudes 0.85) and 0.97 at follow-up in this study. An overall stigma score is calculated by summing the ratings for all 40 items. A 75% cut-off was used for stigma and non-stigma, which was a score of 99.0.

*Sociodemographic factors* assessed included age, education, income, partner status, and number of children.

*HIV-specific issues* assessed included date of HIV diagnosis, a 12-item measure on HIV knowledge (Cronbach’s α = 0.69 and 0.65, respectively, at the prenatal and postnatal assessment points), and an adaptation of the AIDS-Related Knowledge Test. Items reflect information about HIV transmission, reinfection with resistant virus, and condom use knowledge (Carey & Schroder, 2002).

*Partner-specific issues* assessed included disclosure of HIV status to partner, HIV status of partner, consistency of condom use, and an 11-item male involvement index (Jones et al., 2014) (Cronbach’s α = 0.83 and 0.82).

*Intimate partner violence* was assessed using an adaptation of the Conflict Tactics Scale 18 (CTS-18) (Straus, 1979), which included a 9-item partner psychological victimisation subscale (Cronbach alpha 0.76, 0.66, 0.83 and 0.83, respectively, at the two prenatal and two postnatal assessment points), and 9-item partner physical violence subscale (Cronbach alpha 0.92, 0.89, 0.94 and 0.94 at the four assessment points). *Emotional status* was assessed with the Edinburgh Postnatal Depression Scale 10 (EPDS-10) (Cox, Holden, & Sagovsky, 1987). Scores range from 0 to 30, with a validated cut-off score of 12 for South African populations (Lawrie, Hofmeyr, de Jager, & Berk, 1998). Cronbach’s alpha for the EDPS-10 scale ranged from 0.66 to 0.70 at the assessment points in this study.

## Data analysis

Statistical analyses included descriptive statistics (such as means, standard deviations, frequencies and percentages), as well as t-tests or non-parametric tests and chi-square tests. The assumption of normality was violated for weeks of pregnancy, adherence, months since HIV diagnosis, months since ART initiation, and physical intimate partner violence, and as such, Mann–Whitney U tests – a non-parametric alternative to the t-test – were used to compare women not endorsing and endorsing stigma. Therefore, a non-parametric test (Mann–Whitney) was used. Two by two contingency chi-square tests were used to compare women not endorsing and endorsing stigma by categorical tables.

Multinomial logistic regression was used to compare prenatal stigma at baseline with stigma at 12 months postnatally. The dependent variable consisted of women having no prenatal and no postnatal stigma at 12 months (reference category), women with prenatal and postnatal stigma at baseline and 12 months, women who developed stigma from baseline to 12 months, and women who changed from having prenatal stigma at baseline to no longer having postnatal stigma at 12 months. Having stigma was defined as having scored 99 or more on the HIV stigma scale. Condition (intervention versus control) was included in all multivariable analyses, using the control condition as the reference group, regardless of association with the outcome in unadjusted analyses.

Longitudinal analyses were conducted with two assessment points of stigma (model continuously) from prenatal (baseline) to postnatal period (12-months postpartum) as the dependent variable. Two separate models were estimated for time-invariant and time-varying predictors of change in stigma. The time-invariant predictors of stigma over time included age, education, employment status, income, relationship status, pregnancy planning, diagnosed during this pregnancy, months since ART initiation, HIV-positive children, HIV-positive partner, and alcohol use. The time-varying predictors of stigma over time included disclosure of HIV status to partner, male involvement, psychological and physical intimate partner violence, adherence to ARVs, condom use, HIV knowledge, and depression. Condition (intervention versus control) was included as a fixed effect in the time-varying model. Estimated effects are reported with 95% confidence intervals, with missing data accounted for using multiple imputation (Asparouhov & Muthén, 2010). All statistical analyses were performed using Mplus (version 7.4) (Muthén & Muthén, 2014).

## Results

Trial recruitment was conducted from April 2014 to April 2015 and ended in March 2017. From 709 eligible pregnant women identified, eight declined to participate, and two had incomplete data, resulting in 699 patients across 12 community health centres. Participants attended the randomized condition at their community health centres, 342 participants in the EI condition, and 357 participants in the SC condition.

Of N = 699 women who participated at baseline, there were *n* = 16 whose data was lost due to technical difficulties. Of these N = 683 women who completed a baseline assessment, *n* = 445 women returned at 12 months postnatally to complete a follow-up assessment, resulting in a retention rate of 65.2%.

### Sample characteristics at baseline

In all, 699 women living with HIV were enrolled during pregnancy (8–24 weeks) and completed assessments at baseline; 416 (59.5%) women completed assessments at 12 months postnatally. The mean age of the women was 28.4 (SD = 5.8) years, with a range of 18 to 46 years. The majority (78%) had 10 years or more of education, 78% were unemployed, 67% had a monthly income of less than 950 South African Rand ($76), and 40.7% were married or cohabiting. In more than half (53%), the current pregnancy had not been planned. The mean number of weeks of pregnancy at enrolment was 17.8 weeks. More than half (54%) of women were diagnosed with HIV in their current pregnancy, and 81% reported that they had disclosed their HIV status to their partner. Among those women who had children, 5% knew that they had an HIV-infected child, 64% reported their spouse/partner was HIV positive, and 59% of them were on ART. Nearly 1 in 5 women (20%) had not used a condom at their last sexual intercourse (see Table 1). As outlined in Table 1, stigma was associated with alcohol use, depression, decreased HIV knowledge, non-disclosure of HIV status, physical intimate partner violence, psychological intimate partner violence, and decreased male involvement.

**Table 1.** Relationships with Stigma Experience Among HIV Positive Women (*N* = 683).

Characteristic	Groups			Z/t/χ^2^, *p*
	All (*n* = 683) *n*(%) Mean(SD)	Non-Stigma (*n* = 517) *n*(%) Mean(SD)	Stigma (*n* = 166) *n*(%) Mean(SD)	
Sociodemographic				
Age	28.41 (5.78)	28.11 (0.25)	29.32 (0.48)	**−2.23, 0.026^a^**
Educational attainment				
0–10 years	149 (21.8%)	108 (20.9%)	41 (24.7%)	1.58, 0.454
10–11 years	340 (49.8%)	257 (49.7%)	83 (50.0%)	
12 years or more	194 (28.4%)	152 (29.4%)	42 (25.3%)	
Employment status				
Unemployed	533 (78.0%)	401 (77.6%)	132 (79.5%)	0.28, 0.869
Employed	119 (17.4%)	92 (17.8%)	27 (16.3%)	
Volunteering or Student	31 (4.5%)	24 (4.6%)	7 (4.2%)	
Monthly household income (South African Rand)				
<310 (∼ $25)	225 (32.9%)	175 (33.8%)	50 (30.1%)	1.55, 0.462
310–949 (∼ $76)	233 (34.1%)	170 (32.9%)	63 (38.0%)	
950 or more	225 (32.9%)	172 (33.3%)	53 (31.9%)	
Marital status				
Not married, living separate	405 (59.3%)	306 (59.2%)	99 (59.6%)	1.20, 0.550
Not married, living together	153 (22.4%)	120 (23.2%)	33 (19.9%)	
Married	125 (18.3%)	91 (17.6%)	34 (20.5%)	
Alcohol use of 1 or more drinks at least once in the past 4 weeks				
No	589 (86.2%)	454 (87.8%)	135 (81.3%)	**4.46, 0.035**
Yes	94 (13.8%)	63 (12.2%)	31 (18.7%)	** **
Pregnancy unplanned				
No	322 (47.1%)	243 (47.0%)	79 (47.6%)	0.02, 0.895
Yes	361 (52.9%)	274 (53.0%)	87 (52.4%)	
Weeks pregnant	17.81 (5.68)	18.13 (5.69)	16.80 (5.53)	**−2.46, 0.014^a^**
Adherence	6.02 (2.00)	6.82 (1.86)	5.83 (2.18)	−1.44, 0.151^a^
Depression (EDS Score)				
EDS Score is 0–12	349 (51.1%)	282 (54.5%)	67 (40.4%)	**10.12, 0.001**
EDS Score is 13 or greater	334 (48.9%)	235 (45.5%)	99 (59.6%)	** **
**HIV Related Variable**				
Diagnosed during this pregnancy				
No	314 (46.0%)	239 (46.2%)	75 (45.2%)	
Yes	369 (54.0%)	278 (53.8%)	91 (54.8%)	0.06, 0.814
HIV serostatus of children				
Do not know	513 (94.6%)	386 (95.1%)	127 (93.4%)	0.58, 0.448
Positive	29 (5.4%)	20 (4.9%)	9 (6.6%)	
Children currently on ART				
No	2 (6.9%)	1 (4.5%)	1 (14.3%)	FET, 0.79, 0.431
Yes	27 (93.1%)	21 (95.5%)	6 (85.7%)	
Disclosure of serostatus (to anyone)				
No	188 (27.5%)	134 (25.9%)	54 (32.5%)	2.75, 0.097
Yes	495 (72.5%)	383 (74.1%)	112 (67.5%)	
Months since HIV diagnosis	23.45 (36.99)	23.02 (35.72)	24.78 (40.77)	−0.40, 0.688^a^
Months since ART initiation	13.24 (24.32)	12.89 (24.19)	14.31 (24.77)	−0.55, 0.586^a^
HIV Knowledge	13.76 (3.20)	14.06 (3.16)	13.33 (3.22)	**−3.16, 0.002^a^**
**Partner Variable**				
Disclosure of serostatus (to partner)				
No	93 (18.8%)	63 (16.4%)	30 (26.8%)	**6.07, 0.014**
Yes	402 (81.2%)	320 (83.6%)	82 (73.2%)	** **
HIV serostatus of spouse/partner				
Negative/Do not know	97 (36.2%)	83 (38.2%)	14 (27.5%)	2.09, 0.149
Positive	171 (63.8%)	134 (61.8%)	37 (72.5%)	
Partner currently on ART				
No/Unknown	71 (41.5%)	55 (41.0%)	16 (43.2%)	0.06, 0.810
Yes	100 (58.5%)	79 (59.0%)	21 (56.8%)	
Intimate Partner Violence				
No mild / Severe physical violence	547 (80.1%)	423 (81.8%)	124 (74.7%)	**3.99, 0.046**
Mild or severe physical violence	136 (19.9%)	94 (18.2%)	42 (25.3%)	** **
Psychological Intimate Partner Violence	3.23 (5.31)	3.02 (5.24)	3.92 (5.48)	**−2.28, 0.023**
Physical Intimate Partner Violence				
Male involvement index	7.10 (3.07)	7.39 (2.98)	6.20 (3.17)	**−4.33,<0.001^a^**
Adherent to ARVs				
No	224 (32.8%)	162 (31.3%)	62 (37.3%)	** **
Yes	459 (67.2%)	355 (68.7%)	104 (62.7%)	2.06, 0.151

Note*.* ART = Antiretroviral Therapy. FET = Fisher’s Exact test.

^a^Mann Whitney U test was used to compare groups.

### Stigma experienced

To assess the impact of the intervention on HIV-related stigma, the overall and the four factors from Berger’s HIV stigma scale were examined. Baseline scores of overall HIV related stigma and the four scale factors (personalised stigma, disclosure concerns, negative self-image, and concern public attitudes) decreased at follow-up in the intervention group, while baseline scores of overall stigma and three scale factors (personalised stigma, negative self-image, and concern public attitudes) increased at follow-up in the control group (see Table 2).

**Table 2.** Descriptive statistics of HV related stigma.

Stigma dimension		Intervention		Control		P-value
	Time	M	SD	M	SD	
Personalised stigma	1	32.5	10.9	33.5	8.8	<0.001
	2	31.4	10.3	35.3	11.7	
Disclosure concerns	1	19.2	3.9	20.6	4.1	<0.001
	2	18.3	4.4	20.5	4.6	
Negative self-image	1	17.1	4.1	18.1	4.0	<0.001
	2	16.9	4.1	19.0	4.7	
Concern with public attitudes	1	17.6	5.5	18.7	4.6	<0.001
	2	16.8	5.4	19.3	6.1	
Overall	1	86.3	21.8	90.8	18.3	<0.001
	2	83.4	21.8	94.1	24.8	

### HIV stigma change

In multinomial logistic regression analyses, women who were older (AOR = 1.06 [95% 1.00, 1.11]) and those with decreased male involvement (AOR = 0.88 [95% 0.79, 0.96]) were more likely to endorse stigma at both baseline to 12-months postnatally. Emergence of stigmatising attitudes from baseline to 12 months was associated was reported among women of younger age (AOR = 0.94 [95% 0.89, 0.99]), and those in the control condition (AOR = 1.98 [95% 0.99, 3.94]) approached significance. Reduced endorsement of stigma from baseline to 12 months were associated with the intervention condition (AOR = 0.50 [95% 0.31, 0.79]; see Table 3).

**Table 3.** Bivariate and multivariable multinomial logistic regressions with ‘Stable no Stigma’ (prenatal and 12 months postnatal) as reference group (*n* = 222).

	Stable Stigma (*n* = 57)		Change to Stigma (*n* = 63)		Change to No Stigma (*n* = 103)	
	OR [95% CI]	AOR [95% CI]^a^	OR [95% CI]	AOR [95% CI]^a^	OR [95% CI]	AOR [95% CI]^a^
Fixed Effects						
Intervention	0.57 [0.33, 0.98]*	0.63 [0.35,1.05]	0.52 [0.34, 0.79]**	**1.98 [0.99, 3.94]^**	1.32 [0.87, 2.02]	0.50 [0.31, 0.79]**
Covariates (Baseline)						
Age	1.08 [1.04, 1.13]***	1.06 [1.00,1.11]*	0.96 [0.92, 1.00]*	0.94 [0.89,0.99]*	0.99 [0.95, 1.03]	–
Educational Attainment (ref = up to 10 years)						
10 to 11 years	1.29 [0.65, 2.57]	–	0.93 [0.55, 1.57]	–	0.66 [0.39, 1.09]	–
12 years or more	0.99 [0.46, 2.17]	–	0.88 [0.49, 1.59]	–	0.62 [0.35, 1.10]	–
Monthly Income	1.16 [0.69, 1.94]	–	0.95 [0.62, 1.43]	–	0.84 [0.55, 1.28]	–
Relationship Status (ref = unmarried living separate)						
Unmarried, living together	0.87 [0.44, 1.72]	–	0.84 [0.50, 1.42]	–	0.88 [0.51,1.49]	–
Married	1.39 [0.73, 2.63]	–	0.74 [0.41, 1.32]	–	0.99 [0.56,1.72]	
Pregnancy Unplanned	1.04 [0.62, 1.76]	–	1.34 [0.88, 2.04]	–	0.95 [0.62,1.45]	–
Diagnosed during this pregnancy	0.81 [0.48, 1.35]		1.60 [1.04, 2.46]*	1.57 [0.78, 3.15]	1.19 [0.78, 1.83]	–
Months Since ART Initiation	1.01 [1.01, 1.02]**	1.01 [0.99, 1.02]	1.00 [1.00, 1.01]	–	0.99 [0.98, 1.00]*	1.00 [0.99,1.01]
HIV Positive Children	3.02 [1.23, 7.43]*	2.11 [0.67,5.53]	1.14 [0.42, 3.08]	–	0.43 [0.10, 1.85]	–
HIV Positive Partner	0.93 [0.50, 1.70]	–	0.66, 0.39, 1.12]	–	0.79 [0.48, 1.32]	–
Alcohol Use	1.23 [0.60, 2.50]	–	0.63 [0.32, 1.26]	–	1.84 [1.07, 3.15]*	0.71 [0.32, 1.57]
Disclosure of HIV Status to Partner	**0.65 [0.38, 1.09]^**	0.65 [0.31,1.18]	**0.66 [0.44, 1.01]^**	1.18 [0.60, 2.31]	0.65 [0.43, 0.99]*	0.66 [0.38,1.19]
Male Involvement	0.88 [0.81, 0.95]**	0.88 [0.79, 0.96]*	0.97 [0.91, 1.04]	–	0.91 [0.86, 0.98]**	0.96 [0.88,1.05]
Psychological Intimate Partner Violence	1.01 [0.96, 1.05]	–	**1.01 [0.97, 1.04]**	1.03 [0.97, 1.09]	1.04 [1.00, 1.07]*	1.02 [0.95, 1.06]
Physical Intimate Partner Violence	0.99 [0.92, 1.07]	–	1.00 [0.94, 1.06]	–	1.09[1.04, 1.14]***	1.04 [0.95, 1.14]
Depression	1.02 [0.61, 1.71]		0.791 [0.52, 1.20]	–	2.27 [1.46,3.52]***	0.90 [0.57, 1.43]
Adherence	1.21 [1.00, 1.47]*	1.17 [0.95, 1.40]	1.03 [0.93,1.15]	–	0.88 [0.81, 0.96]**	1.03 [0.91, 1.16]
Model Fit						
-2LL (Deviance)	−144.96		−102.83		−246.47	
Number of Parameters	8		6		10	
AIC/BIC	305.93/337.28		217.66/236.29		512.94/555.27	

Note. AOR = Adjusted Odds Ratio.

^a^The odds ratio for each independent variable is adjusted by each of the other variables in the model.

***P < 0.001.

**P < 0.01.

*P < 0.05.

^*P* < 0.10.

### HIV stigma outcomes

Results of longitudinal analyses of stigma outcomes are presented in Table 4. Using longitudinal analyses, Model 1, which included time-invariant predictors of stigma assessed over the two time periods of baseline and 12 months, increases in stigma from baseline to 12 months were associated with being unemployed (b = 1.449 [95% CI 0.33, 2.56]), having been diagnosed with HIV before the current pregnancy (b = 1.19 [95% CI 0.83, 2.30]), and alcohol use (b = −1.19 [95% CI −2.34, −0.027]). In Model 2, which included time-varying predictors, lower stigma scores were associated with participation in the intervention (b = −1.39 [95% CI −2.13, −0.65])., greater male partner involvement (b = −1.29 [95% CI −1.85, −0.73]), and consistent condom use (b = −0.42[95% CI −3.06, 2.22]).

**Table 4.** Predictors of HIV related stigma at baseline and 12-months postnatally.

Variable	Coefficients (95% CI)
Model 1: Baseline characteristics (time-invariant)	
Age	0.020 [−0.079, 0.119]
Education	
0-Grade 9	1 (Reference)
Grade 10–11	0.430 [−0.609, 1.469]
Grade 12 or more	−0.386 [−1.659, 0.886]
Employed	
No	1 (Reference)
Yes	1.449 [0.333, 2.564]*
Income	
600 or more	1 (Reference)
<600 Rand	−0.876 [−1.801, 0.050]
Relationship status	
Unmarried, living separate	1 (Reference)
Unmarried, living together	−0.334 [−1.525, 0.857]
Married	0.841[−0.335, 2.016]
Pregnancy unplanned	
No	1 (Reference)
Yes	0.213 [−0.666, 1.091]
Diagnosed during this pregnancy	
No	1 (Reference)
Yes	1.190 [0.083, 2.296]*
Months since ART initiation	0.000 [−0.017, 0.017]
HIV positive children	
No	1 (Reference)
Yes	0.064 [−1.546, 1.675]
HIV serostatus of spouse/partner	
Negative/Do not know	1 (Reference)
Positive	−0.459 [−1.559, 0.641]
Alcohol (>2 drinks last month)	
No	1 (Reference)
Yes	−1.185 [−2.343, −0.027]*
*Random Effects^a^*	
Intercept (baseline)	−0.069 [−2.264, 5.227]
Stigma	16.60 [−3.121, 2.984]
Model 2: Variables assessed at two assessments (time-varying)	
Fixed Effects	
Intervention	
Standard of care	1 (Reference)
Enhanced intervention	−1.391 [−2.128, −0.653]***
Disclosure of HIV Status to Partner	0.839 [−2.312, 3.989]
Male Involvement	−1.289 [−1.853, −0.725]***
Psychological Intimate Partner Violence	0.492 [−1.195, 2.180]
Physical Intimate Partner Violence	2.175 [−0.153, 4.504]
Adherence to ARVs	2.485 [−3.293, 8.264]
Consistent condom use (past week)	−0.418 [−3.055, 2.219]*
Noncondom use at last sex	−3.798 [−6.697, −0.899]
HIV Knowledge	0.250 [−0.367, 0.867]
Depression	2.293 [−0.364, 4.949]
Random Effects^a^	
Intercept (baseline)	10.246 [8.154, 12.339]***
Stigma	12.764 [10.836, 14.693]***

CI = Confidence Interval.

^a^Random effects indicates the estimated variances from random effects logistic regression model.

***P < 0.001.

**P < 0.01.

*P < 0.05.

## Discussion

The study examined the impact over time of a multi-session cognitive behavioural PMTCT intervention, including stigma, on HIV-related stigma among HIV-infected pregnant women. The current study addressed HIV-related stigma within the contextual of partner communication, HIV status disclosure and medication adherence, resulting in a reduction in HIV related stigma. Results are unique; the only other stigma intervention trial among HIV-infected women found decreases in stigma did not differ between intervention and control conditions (Barroso et al., 2014). Additionally, the current study identified reductions in all aspects of stigma assessed (personalised stigma, disclosure concerns, negative self-image, and concerns regarding attitudes of the public) in intervention participants and concomitant increases in control participants at long term follow up, in contrast with previous stigma interventions that achieved only a reduction in negative self-image was maintained at 3 months follow-up (Harper et al., 2014).

Results suggest that the current intervention may have strengthened resistance to stigma that may have impacted both internal and external perceptions of HIV-related stigma (Harper et al., 2014). Resistance may have been supported by the group sessions, which focus on decreasing negative feelings about living with HIV, planning for HIV status disclosure and skill building to resist HIV-related stigma. This is supported by the observed HIV-related stigma reductions in personalised stigma (experiences or fears of rejection because of having HIV), disclosure concerns (keeping HIV status to themselves or being in control of disclosure), negative self-image (having negative feelings because of the fact of having HIV), and concern with public attitudes, such as discrimination about their HIV positive status. As noted, stigma reduction programmes appear to have increased effectiveness when they include multiple sessions (Mak et al., 2017). Thus, it is possible that stigma reduction programmes such as ‘Protect Your Family’ could have even greater benefit if the stigma component were to be expanded to more than the first two sessions.

Between the four components of HIV-related stigma, disclosure concerns ranked highest(item mean 2.46), followed by personalised stigma (M = 2.33), concerns with public attitudes (M = 2.22) and negative self-image (M = 2.18) at study entry and long term follow-up. A similar finding was obtained among young people, who indicated they were most concerned about HIV disclosure. The predominant concern with HIV status disclosure the study population of perinatal HIV-infected women also supports previous qualitative research by this team in rural South Africa (Mlambo & Peltzer, 2011; Shikwane, Villar-Loubet, Weiss, Peltzer, & Jones, 2013).

Finally, lack of perinatal male involvement and inconsistent condom use was associated with higher HIV stigma. Unlike previous studies public stigma attitudes (Li et al., 2010; Mak et al., 2017), there was no relationship between HIV knowledge and HIV related stigma. In fact, older age was related to sustained stigma, while emergence of stigmatising attitudes was associated with younger age. This may be due to an increasing difficulty among older persons to change stigmatising attitudes, while for younger women, new experiences of HIV stigma could have led to the development of perceptions of HIV-related stigma.

## Study limitations

The study experienced high rates of loss to follow-up, which may have limited the generalizability of the study outcomes, and may reflect more active participation by those experiencing less stigma. This outcome should, however, be limited by randomisation.

## Conclusion

This study examined the longitudinal experience of stigma, and the impact of an enhanced PMTCT intervention, including stigma reduction, on stigma outcomes. The intervention, which was administered by trained lay health workers, had a significant effect on the reduction of HIV related stigma. Future interventions to reduce HIV related stigma among perinatal women could build on the stigma reduction components utilised in the intervention and expand them to develop a more comprehensive stigma reduction programme appropriate to the South African context.
